# Ex-Ante Economic Impact Assessment of Genetically Modified Banana Resistant to *Xanthomonas* Wilt in the Great Lakes Region of Africa

**DOI:** 10.1371/journal.pone.0138998

**Published:** 2015-09-28

**Authors:** John Herbert Ainembabazi, Leena Tripathi, Joseph Rusike, Tahirou Abdoulaye, Victor Manyong

**Affiliations:** 1 International Institute of Tropical Agriculture (IITA), Kampala, Uganda; 2 International Institute of Tropical Agriculture (IITA), Nairobi, Kenya; 3 International Institute of Tropical Agriculture (IITA), Dar es Salaam, Tanzania; 4 International Institute of Tropical Agriculture (IITA), Ibadan, Nigeria; 5 Alliance for a Green Revolution in Africa (AGRA), Nairobi, Kenya; Dong-A University, REPUBLIC OF KOREA

## Abstract

**Background:**

Credible empirical evidence is scanty on the social implications of genetically modified (GM) crops in Africa, especially on vegetatively propagated crops. Little is known about the future success of introducing GM technologies into staple crops such as bananas, which are widely produced and consumed in the Great Lakes Region of Africa (GLA). GM banana has a potential to control the destructive banana Xanthomonas wilt disease.

**Objective:**

To gain a better understanding of future adoption and consumption of GM banana in the GLA countries which are yet to permit the production of GM crops; specifically, to evaluate the potential economic impacts of GM cultivars resistant to banana Xanthomonas wilt disease.

**Data Sources:**

The paper uses data collected from farmers, traders, agricultural extension agents and key informants in the GLA.

**Analysis:**

We analyze the perceptions of the respondents about the adoption and consumption of GM crop. Economic surplus model is used to determine future economic benefits and costs of producing GM banana.

**Results:**

On the release of GM banana for commercialization, the expected initial adoption rate ranges from 21 to 70%, while the ceiling adoption rate is up to 100%. Investment in the development of GM banana is economically viable. However, aggregate benefits vary substantially across the target countries ranging from US$ 20 million to 953 million, highest in countries where disease incidence and production losses are high, ranging from 51 to 83% of production.

**Conclusion:**

The findings support investment in the development of GM banana resistant to Xanthomonas wilt disease. The main beneficiaries of this technology development are farmers and consumers, although the latter benefit more than the former from reduced prices. Designing a participatory breeding program involving farmers and consumers signifies the successful adoption and consumption of GM banana in the target countries.

## Introduction

Investments in biotechnology development are leading to the introduction of genetically modified (GM) crops with significant impacts on yield improvement, poverty reduction and food security. These impacts have been assessed using ex post and ex ante analyses [1–11]. Finger et al. [12] and Klümper and Qaim [13] demonstrated impressive impacts from GM crops: a significant reduction in pesticide use, yield gains and increased profits for farmers. Genetic modification of staple food crops such as banana and rice for resistance to diseases, pests and abiotic stresses can be expected to substantially alleviate poverty, hunger and malnutrition [10, 14]. Farmers have widely adopted the GM crops currently on the market including primarily: cotton, maize, canola and soybean. For example, 170 million hectares, approximately 12% of the global arable land, were planted with GM crops in 2012 [15]. However, contentions about the social implications of GM crops and the resultant conflict among the interests of consumers, producers and the agri-business private sector continues to persist [16]. In addition, GM crops have been bred and produced in countries which have embraced laws on biosafety and biotechnology policies. In Africa, Cabanilla et al. [17] demonstrated high economic returns from investing in Bt-cotton in West Africa. What remains unanswered is whether the introduction of GM technologies into crops which are widely consumed by both farmers and urban dwellers will be successful, especially in countries which are yet to permit their production. This paper responds to this question by evaluating the potential economic impacts of GM cultivars resistant to banana *Xanthomonas* wilt (BXW) disease in the Great Lakes region of Africa (GLA).

The GLA region comprising of Kenya, Tanzania, Uganda, Burundi, Rwanda, and the eastern part of the Democratic Republic of Congo (DRC), is the largest producer of banana in Africa. The metrics computed by the authors from Food and Agriculture Organization (FAO) online data show that 60% of the total area under banana cultivation across Africa is in the GLA, with 63% of Africa’s production and 21% of the world’s banana supply [18]. At the same time, the GLA is the highest consumer of its banana in the world with 3–22% of total calorie consumption per capita, and 147 kcal daily consumption per person, which is 15 times the world’s average and 6 times Africa’s average [18]. Banana is not only a staple food in the GLA region but also an important source of cash income. Increasing the productivity and profitability of production are important steps in achieving household and national food security and driving down the real price of food to accelerate economic growth, increase the sustainable management of natural resources, improve nutrition and health and reduce poverty.

However, banana production is being seriously threatened by the outbreak and spread of BXW [19] caused by *Xanthomonas campestris* pv. *musacearum* (Xcm). BXW affects both the quantity and quality of fruits. Crop losses can be as high as 100%. Currently, two major approaches exist to control the disease. First, the use of cultural practices, which involves removing the male bud (to prevent infection carried by insects), using sterilized farm tools and destroying single infected stems (or the whole mat). However, the level of BXW control by cultural practices can be inconsistent when the value chain actors, especially the farmers and traders, fail to comply in implementation. The second approach would be to use natural host plant resistance to prevent the disruptive impacts of BXW but this is non-existent in the germplasm of cultivated banana. In the circumstances, the International Institute of Tropical Agriculture (IITA) in partnership with the National Agricultural Research Organization (NARO) of Uganda and African Agricultural Technology Foundation (AATF) has successfully developed GM varieties resistant to BXW. The transgenic varieties were developed through constitutively expressing Hypersensitive Response Assisting Protein (*Hrap*) or Plant Ferredoxin Like Protein (*Pflp*) gene from sweet pepper (*Capsicum annuum*) [20, 21]. The wilt-resistant genes extracted from pepper are not listed as a potential allergen in AllergenOnline and should be safe for human consumption. These proteins are widely distributed across a broad range of plant species including vegetables that are, like sweet pepper, eaten raw. Importantly, the risk of gene escape to other crops is unlikely since most edible bananas are sterile and the process involves clonal propagation which limits gene flow to other crops.

The GM banana plants have been evaluated in Uganda and have shown absolute resistance to Xcm [22, 23]. The transgenic lines showed flowering and yield characteristics comparable to non-transgenic varieties and are currently under evaluation for the durability of disease resistance and agronomic performance in the second field trial in Uganda. The identified lines will be further evaluated in multi-locations in Uganda for environmental and food safety in compliance with the country’s biosafety regulations. The best lines will then follow the procedures for risk assessment and management, seed registration and release. The GM banana is likely to be released for multiplication, distribution and commercialization in 2020. Evidence exists on the technical performance of the new technology against BXW as explained above, but no empirical data have documented its economic profitability and potential acceptability.

This study assesses the potential impact of the GM varieties on the economic benefits of production prior to their release, dissemination and commercial sale to farmers. Specifically, the study’s objectives are two-fold. First, to assess production and consumption patterns, estimate a baseline scenario for production and marketing and forecast the likely changes with adoption of GM banana. Second, to evaluate the potential benefits and costs of GM banana to producers and consumers. The findings show that both producers and consumers would benefit. However, the latter would benefit more than the former since high production levels drive down consumer prices.

## Materials and Methods

The study used rapid appraisal value chain analysis and ex-ante benefit-cost framework utilizing the economic surplus model (ESM). The value chain framework was used to collect and analyze data on production and consumption and the identification of geographical areas and varieties for potential intervention. The ESM was used to estimate the economic benefits of developing GM bananas resistant to BXW (GMB-BXW) but because the GM bananas have not yet been commercially released, it is not possible to measure the impacts under farmers’ conditions and their acceptability to consumers. However, the ESM, described in detail below, has the ability to quantify the potential benefits and costs of GMB-BXW accruing to consumers and farmers.

### Data Sources

The study was conducted in six countries in 2013: Burundi, DRC, Kenya, Rwanda, Tanzania, and Uganda and was based on major banana-producing areas and the incidence of BXW. Three districts (or an equivalent administrative unit) were selected in each country. Each of the districts represented the regional block in each country. A structured questionnaire was used to collect data from extension agents, key informants, farmers and traders.

The identification and recruitment of respondents followed a purposive sampling procedure. In each country, the selection of respondents started with visits to National Agricultural Research Institutions (NARI) with the help of IITA scientists. At NARI, a resource person was identified to select the three major banana-producing districts and to provide expert opinions as a key informant. In each district, the district agricultural officer or extension agent was identified, first, to participate as a respondent to provide expert opinions. Second, to select villages and markets to include in the study. Third, to identify and select contact persons in the selected villages and markets. These contact persons at the village and market levels helped to identify and select farmers and traders to participate in the study, based on agreed criteria described in the results below.

The consent of each respondent to participate in the study was sought orally; the objectives of the study were explained and a summary of the required information was provided before the interview was administered. Each respondent was informed as follows: participation is voluntary, the data collected will be analyzed and reported anonymously, and the information collected will be used to represent the views of other stakeholders. Then a respondent was asked whether he or she agreed to participate or not. The response was recorded on the questionnaire as ‘YES’ or ‘NO’. The questionnaire and oral consent were reviewed and approved by the head of the IITA’s social science research group and retrospectively by the IITA Institutional Review Board (IITA IRB). The study began before IITA IRB was instituted. All sampled respondents consented to participate and the data were analyzed anonymously to protect confidentiality. The data are freely available online (S1 Dataset). Table 1 reports the characteristics of respondents. Since we used a rapid appraisal value chain approach to collect data, the interpretation of results should be treated within the spirit of data based on expert opinion and a limited representation of smallholder farmers. Despite this limitation, our results from the surveys are comparable with those found in studies that have used large and representative sample sizes or experimental data from countries under study [24].

**Table 1:** Characteristics of respondents (percentages and means).

Farmers	(N = 37)
% of smallholder farmers (versus progressive)	64.9
% of male respondents	75.7
Average age (years)	55.4
Education (years of schooling)	7.4
Experience in banana production (years)	26.7
% of crop income from bananas	62.2
Traders	(N = 47)
% of banana retail traders (versus wholesalers)	61.7
Experience in agricultural produce trade (years)	14.1
Experience in marketing of bananas (years)	12.2
Number of banana bunches sold on a market day	86.3
% of business income from banana trade	57.1
Agricultural extension agents	(N = 21)
% of extension agents working with government institutions	33.3
Experience in area of academic training (years)	11.5
Experience in banana extension work (years)	10.1
Key informants	(N = 7)
% of key informants working with government institutions	71.4
Experience in area of academic training (years)	16.2
Experience in banana extension work (years)	14.6

Farm level data were collected on production, BXW incidence and its effects on production, potential adoption of GMB-BXW, choice of banana varieties, consumption and marketing from at least 6 farmers in each country. Out of 37 farmers, 65% represented smallholders and 35% represented progressive or model farmers. Progressive farmers were defined as those who were considered by the community to be the largest producers compared with others and generally used improved agricultural practices. Most smallholder farmers were leaning on subsistence production; progressive farmers usually produced banana for sale.

Market information on consumer preferences and attributes for banana was collected from at least 4 retailers and 2 wholesalers from each country, giving a total of 47 traders (29 retailers and 18 wholesalers). Retailers included traders in towns in the study area and itinerant traders in villages where the selected farmers lived. Wholesalers were mainly those supplying banana to major district towns.

Extension agents provided a broad overview of the incidence of BXW, its impacts on production, and the potential adoption of GMB-BXW. The majority of extension agents (66.7%) interviewed worked for international or local non-governmental organizations. In addition to information similar to that collected from extension agents, key informants provided more insight on the spread of BXW countrywide and issues related to policy on biotechnology. At least one key informant and 3 extension agents were interviewed from each country. The key informants included one of the following: senior agricultural government officials, banana breeders and officials from private tissue culture laboratories.

### Analytical approach

There are several approaches to evaluate the ex-ante impact of agricultural technologies including ESM, benefit-cost analysis and econometric models [25]. For this study, the ESM was used. It is preferred to other approaches when acceptable assumptions are used despite its drawbacks to control for measurement error, general equilibrium effects, transaction costs and externalities. Unlike the benefit-cost and econometric analyses, the ESM does not assume perfectly elastic or inelastic demand and supply and it controls for both international prices and distributional effects [25].

For ex-ante analysis in this study, the ESM model for the closed economy was used to estimate the economic benefits for each country due to a change in banana supply resulting from the introduction of GMB-BXW. The details of the formulation to estimate ex-ante economic benefits (producer and consumer surpluses) for a given technology are provided in Alston et al. [25], and these details are not repeated here.

The release and the eventual adoption of GMB-BXW are expected to increase yields substantially from the baseline of the BXW present scenario (i.e., a downward shift in the supply curve), while the demand level remains unchanged. This may lead to a fall in prices. As a result, consumers gain through paying less for more and farmers may benefit through larger supplies to the market. In other words, from the release and adoption of GMB-BXW, both the consumer and producer surpluses may increase leading to increased total economic surpluses (benefits) resulting from the release and adoption of GMB-BXW.

The changes in economic surpluses are measured for the research period from 2013 to 2037 for which tangible research outputs are, and will be, realized. By 2013, researchers had successfully developed and tested transgenic cultivar lines resistant to BXW. This means that research costs prior to 2013 are considered as unrecoverable since these were invested in the acquisition of equipment and in building a knowledge base, which are more or less fixed. The period from 2013 to 2020, the expected release date, is associated with the investments in multi-location fields and biosafety testing. The annual supply changes are then estimated based on the projected adoption rate for GMB-BXW for the period from 2020 to 2037. Costs associated with the projected adoption rate would represent the extension costs for the period following the official release of GMB-BXW. For each country *i* at time *t*, the net present value (NPV) is used to evaluate the changes in economic surplus (*ES*) for the period 2013–2037 as:

$$NPV=\sum_{t=2013}^{2037}\frac{\Delta ES_{i,t}}{{\left(1+r\right)}^{t}}-\sum_{t=2013}^{2037}\frac{C_{i,t}}{{\left(1+r\right)}^{t}}$$ (1)

where *r* is the real discount rate set at 10% per annum, and *C*_*t*_ is the total research cost.

To measure returns on investment into the development of GMB-BXW, the internal rate of return (IRR) was computed. The benefit-cost ratio (BCR) was also calculated to measure returns from each dollar invested in development of GMB-BXW. The BCR is computed as the ratio of NPV of benefits to the net present value of research and extension costs.

## Results and Discussion

### BXW awareness, spread and impact on banana production

**Fig 1:**
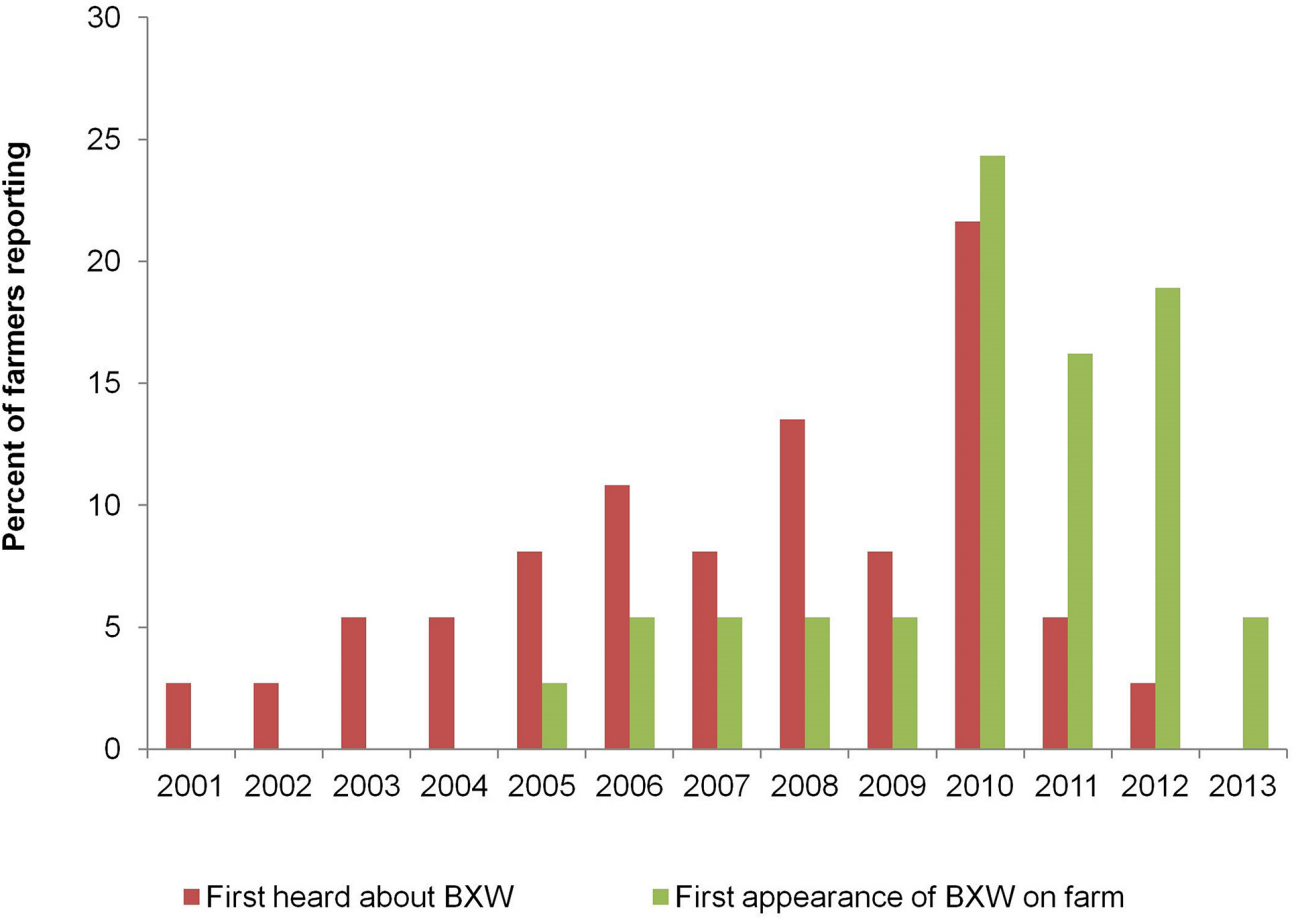
Farmers’ awareness of BXW disease in target countries.

**Fig 2:**
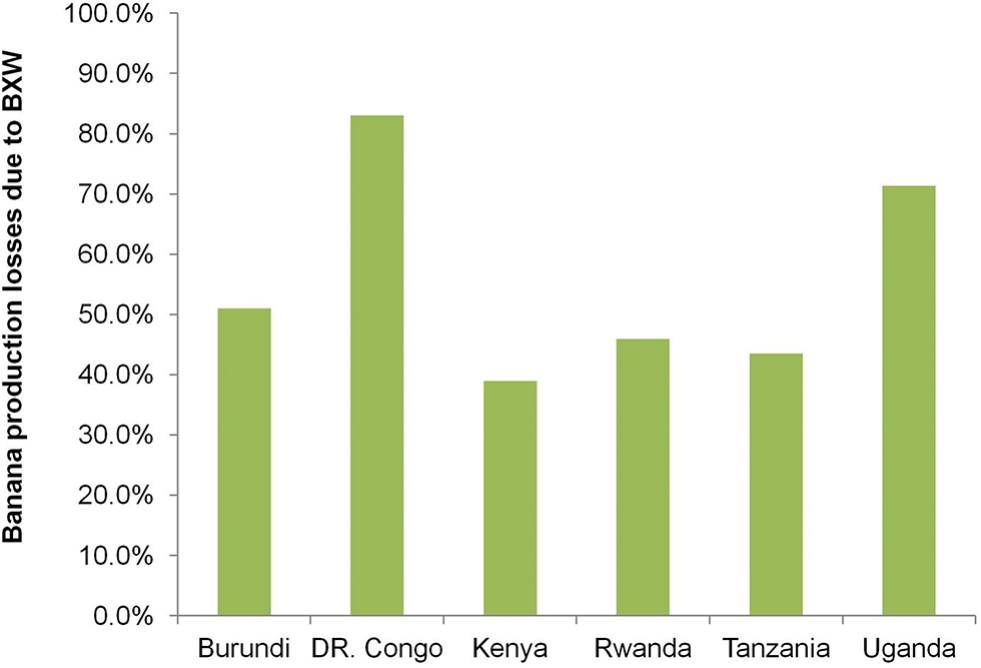
Losses in banana production due to BXW incidences in target countries.

Participating farmers became aware of BXW as far back as 2001, but the majority realized its devastating consequences as recently as 2010 (Fig 1). Although farmers in the sample first heard about BXW around 2001, the first occurrence of BXW on the farmers’ plantations in the study areas generally started in 2005 with the majority experiencing it in 2010 through 2011. The impact on production in the study areas is devastating. The average production losses are highest in DRC (83%) and Uganda (71%), and range from 39 to 51% in other countries (Fig 2). These figures were obtained as averages from farmers, extension agents and key informants. These losses are within the range reported in earlier studies in the region. For example, Karamura et al. [26] estimated in Uganda a loss in banana production of 65–80% due to BXW. The implication is that the existing cultural control methods appear somewhat ineffective in combating the effects of BXW. In Uganda, for example, the first occurrence of BXW was observed in 2001, but production losses have remained high despite the application of existing recommended control methods and a wide mass media campaign by the extension service programs. Results (Table 2) show that more than half of the farmers (54%) used a combination of control methods including removing male buds and infected plants and using sterilized tools, but only 3% avoided the introduction of suckers from unknown locations. This potentially leads to a vicious cycle of BXW occurrence on farmers’ fields—in the sense that, after all infected plants or mats have been removed; the new planting material remains a potential source of infection.

**Table 2:** Control methods for BXW and awareness of genetically modified (GM) crops.

*% of farmers using control methods for BXW (N = 37)*	
Removing the male bud	10.8
Removing infected plants	21.6
Uprooting whole infected mat	18.9
Avoiding introduction of suckers from unknown locations	2.7
Combination of control methods	54.1
% of respondents aware of the development of BXW resistant banana varieties (N = 111)	27.9
% of respondents aware of the meaning of GM banana (N = 75)	36.0
*% of respondents defining GM banana as … (N = 29)*	
a banana which has been bred to resist diseases	34.5
an improved banana with integrated gene(s) from other sources	41.4
a banana variety with different properties from local varieties (good eye appeal but tasteless, has long term health effects)	24.1
*% of respondents’ opinion on the development of GMB-BXW (N = 112)*	
Support the development	83.0
Indifferent	3.6
Do not support the development	13.4
% of respondents reporting potential benefits of GM banana resistant to BXW (N = 111)	94.6
*Perceived potential benefits (N = 217)* (%)	
Increase in yields	28.1
Increase in income	38.3
Credit to research institutions	3.7
Stable supply	12.4
Increase in banana consumption	17.5
% respondents reporting potential disadvantages of GM banana resistant to BXW (N = 110)	40.0
*Perceived potential disadvantages (N = 54)* (%)	
High cost of establishing new plantations	24.0
Undesirable consumption attributes	14.8
Outbreak of new diseases and loss of local varieties	27.8
Health problem concerns	24.1
Failure of GM banana to adapt to local conditions	9.3

Note: In some cases, the number of observations is the sum all respondents in the study (farmers, traders, extension agents and key informants). The number of observations varies for some questions due to missing responses.

### Awareness of GM crops and development of GMB-BXW

For more than two decades, GM crops have been widely grown and are considered safe in a number of countries outside the GLA [12, 13]. It was necessary to gain an understanding of the stakeholders’ perception about the future introduction of GM banana in the target countries. Key informants, extension agents and traders were asked to define a GM banana. The focus on these key stakeholders as change agents was because they have the capacity to cause a behavioral change among farmers through capacity building and increased market demand. About 35% of the respondents who believed they knew the meaning, thought a GM banana was “a banana which has been bred to resist pathogens that cause diseases,” and 41% said it was “an improved banana which has been developed with different genes from other sources.” Others, largely traders, defined the GM banana as “a variety with attributes different from those of the traditional varieties” (Table 2). Some of the attributes mentioned included nice-looking fingers, not being tasty and having long-term health effects on humans. These definitions reflect a knowledge gap in information sharing between research scientists in the breeding profession and the end-users of their products. This suggests that successful adoption of GM crops would require the scientists’ effort to develop and disseminate simple user-friendly manuals describing the development process of the GM crop and its biosafety effects. Alternatively, a participatory approach tailored to breeding programs—in which relevant stakeholders are actively involved in the process—may be appropriate.

Regarding the awareness of the research development on GMB-BXW, nearly one-third of the respondents had heard about GMB-BXW through seminars and colleagues. To enrich the understanding of the respondents about GM crops, an explanation was provided to each of them about the process of developing GMB-BXW. The majority of respondents (83%) supported the development; 4% were undecided and 13% did not support it. However, the majority (95%) reported a number of potential benefits associated with the development of GMB-BXW including increased yields and income, stable and continued supply and increased consumption.

The majority of the respondents (60%) did not perceive any potential disadvantages associated with GMB-BXW development, but 40% reported otherwise. The most important potential disadvantages included the following: the GM banana may lead to the outbreak of new diseases through mutation, which may lead to major losses of local varieties and establishing new plantations incurs a high cost. This is likely to slow down early adoption of GM banana since farmers have already experienced significant income losses from BXW effects and may be unable to establish new plantations or even to afford the BXW-resistant planting materials that are likely to be expensive. The perception that GMOs are not good for human health was frequently mentioned. Other anticipated disadvantages included undesirable attributes, which may affect market demand negatively, and the failure of GM banana to adapt to local environmental conditions, which may lead quickly to the loss of resistance to BXW. For the successful adoption of GMB-BXW, all these misperceptions of potential disadvantages need to be addressed during its dissemination.

### Potential adoption of GMB-BXW

**Fig 3:**
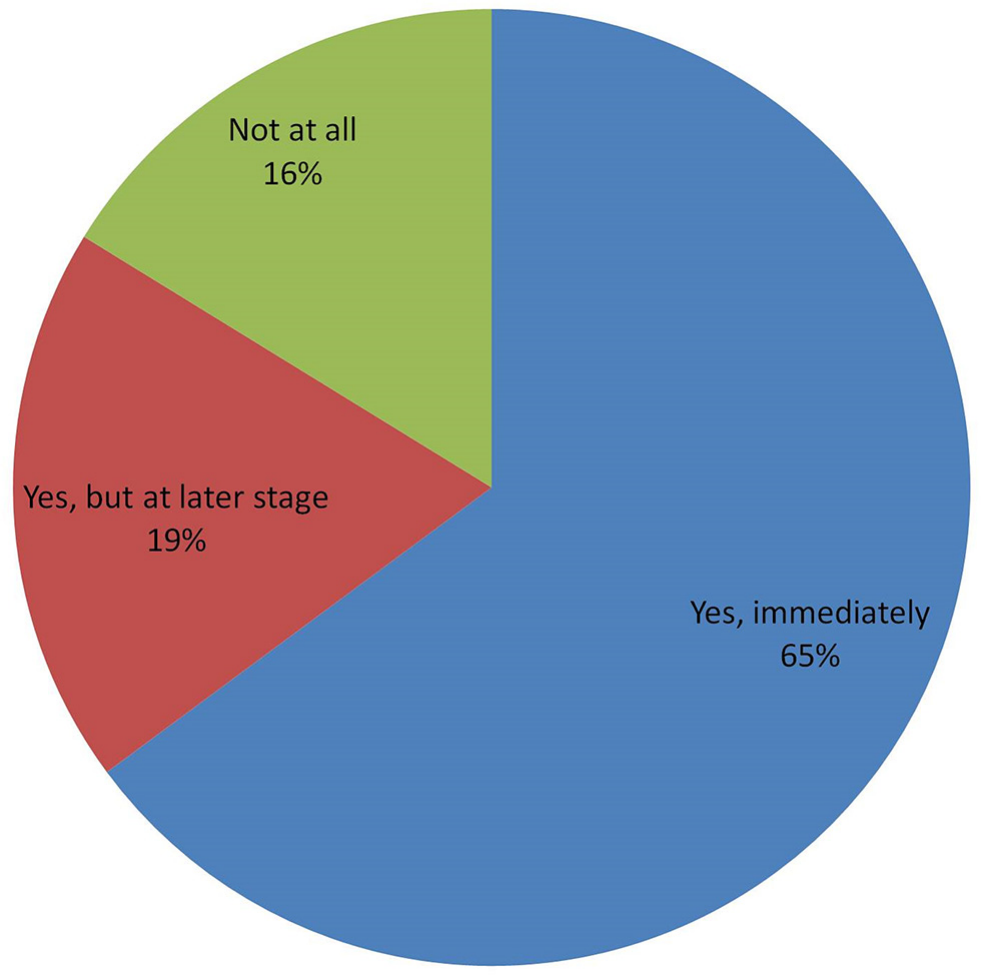
Willingness of farmers to adopt GMB-BXW in target countries.

Fig 3 reports the potential adoption of GMB-BXW among the sample farmers. The majority (65%) stated they intended to adopt GMB-BXW immediately upon release to limit the spread of BXW, restore the destroyed plantations and improve declining crop income through increased yields (Table 3). Other farmers (19%) indicated that they would delay adoption to learn first about the effects and performance of GMB-BXW. Interestingly, nearly all farmers (90%) were willing to pay for GMB-BXW planting material if they decided to adopt it. At the time of the survey, the average market price for a non-GM plantlet was US$ 0.80; some farmers were willing to pay for a discounted GMB-BXW plantlet by 11% relative to the market price, while others were willing to pay price premiums up to 75% of the market price. About 2 acres, on average, would be allocated to GMB-BXW for cultivation. Part of this land would come from cutting down existing plantations already affected by BXW as reported by 27% of the farmers; the majority (73%) reported that they would establish new plantations by reallocating land from other crops.

**Table 3:** Farmers’ willingness to pay for and reasons for adoption of GMB-BXW.

Reasons for early adoption of GMB-BXW (N = 33) (%)	
To limit the spread of BXW	36.4
To restore plantations destroyed by BXW	36.4
To improve yields and income	24.2
To be among early adopters	3.0
Reasons for late adoption of GMB-BXW (N = 9) (%)	
To limit the spread of BXW	22.2
Need to first learn from early adopters	66.7
Negative attitude to improved varieties	11.1
% of farmers willing to pay for GMB-BXW (N = 31)	90.3
Minimum price farmers are willing to pay (US$)	0.71
Maximum price farmers are willing to pay (US$)	1.43
Area expected to be allocated to GMB-BXW (acres) (N = 37)	1.7
% of respondents reporting to cut down existing banana plantation and replace with GMB-BXW (N = 26)	26.9
% of respondents reporting to establish new banana plantation by land re-allocation (N = 26)	73.1

Note: The number of observations varies due to missing responses

**Fig 4:**
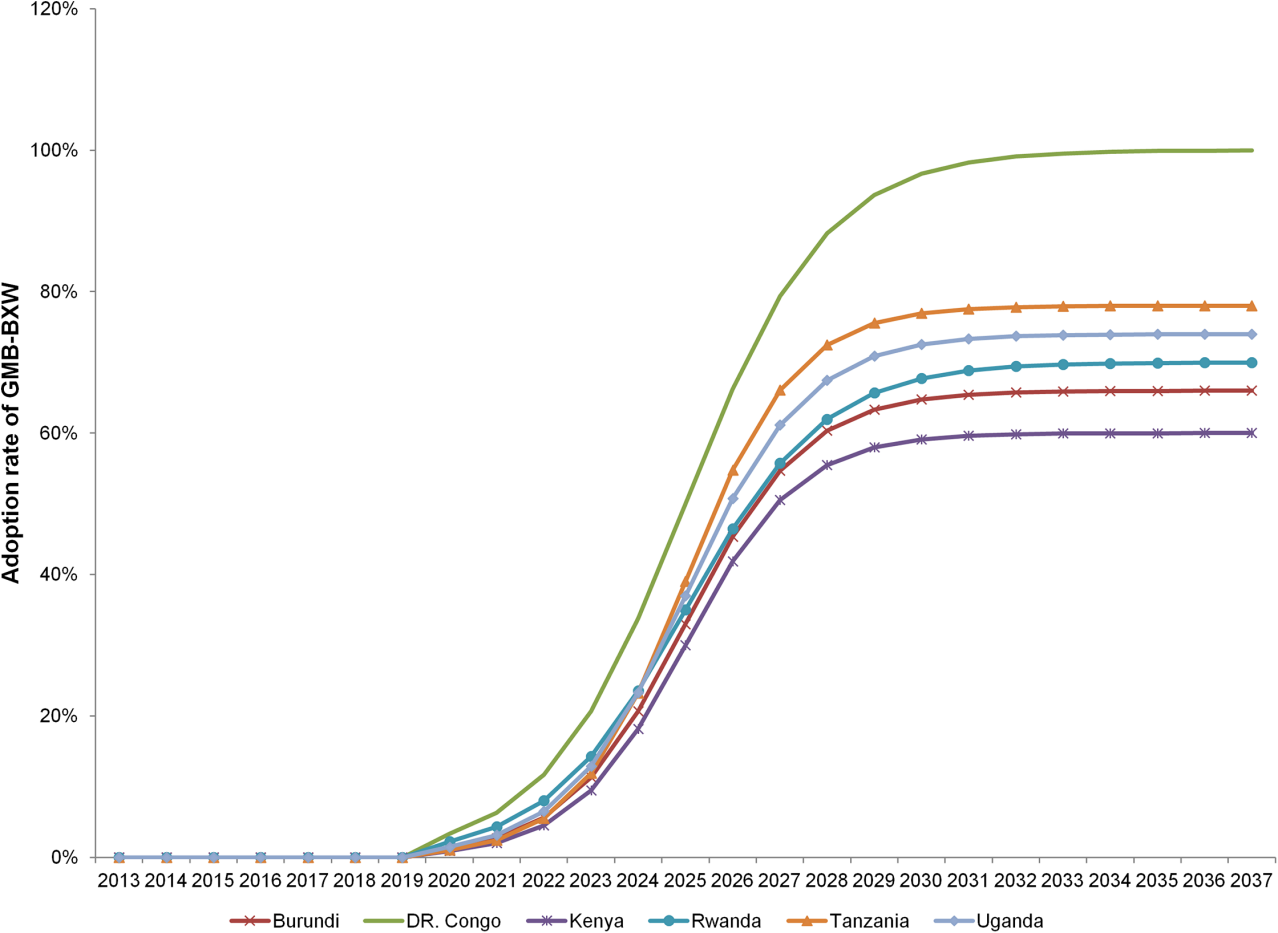
Projected adoption rate of GM BXW resistant banana in target countries.

Extension agents and key informants were also asked to estimate the potential adoption rate based on their experience with the adoption of improved banana varieties. The survey results show that if GMB-BXW is released in 2020, the expected minimum adoption rates are impressively high ranging from 21% in Kenya to 70% in DRC (Table 4). The maximum adoption rates of up to 100% would be attained in 2–10 years from the time of adoption. Fig 4 reports an adoption path for each country predicted over 25 years using the analytical approach suggested by Alston et al. [25]. The first segment of zero adoption indicates the period of field trials and biosafety tests from 2013 up to 2020, the expected year of release.

**Table 4:** Parameter values used to estimate the economic benefits of GMB-BXW using ESM.

Country	Burundi	DRC	Kenya	Rwanda	Tanzania	Uganda
Price per ton (US$)^1^	267	255	198	205	203	207
Research costs (million US$)^2^	3.2	3.2	3.2	3.2	3.2	3.2
*Losses due to BXW and adoption of GMB-BXW* ^3^						
Average loss of banana production due to BXW^a^ (%)	51	83	39	45.9	43.5	71.4
Expected initial adoption rate of BXW resistant banana (%)	30	70	20.9	48	24	35.3
Expected ceiling of adoption rate of BXW resistant banana (%)	65.5	100	60.4	70	77.5	73.8
Expected number of years to attain maximum adoption	3–5	3–5	5–10	2–5	5–10	5–10
Average % increase in area allocated to banana after adoption^a^	23.1	50	30.6	26	10.3	40
Average % increase in input costs per ha after adoption	27.5	50	28	35	20	33
Average % yield gain after adoption per ha	56.7	70	39.4	30	25	53.8
% of banana production from study areas with respect to whole country^a^	15	13	27	38	33	28
*National banana production* ^4^						
Production (‘000 tonnes)	1,184	832	1,424	3,220	3,260	9,770
Banana area (ha)	178	361	61	349	727	1,830

Data sources

^1^The average farm gate price from the 37 surveyed farmers who sold bananas.

^2^Research costs extracted from the GMB-BXW project proposal budget for Kenya and Uganda.

^3^Key informant survey data (from banana breeders, research scientists and extension agents). Key informants were asked to provide information on banana losses, potential adoption of GMB-BXW, and production by major regions producing bananas in their respective countries. The figures provided are average figures across regions in each country and the sample sizes vary per country.

^4^FAOSTAT for data for the year 2014, available on http://faostat.fao.org.

^a^Note that these variables were not used in the ESM, but are reported to provide additional explanation.

### Consumption preferences: local versus improved varieties

**Fig 5:**
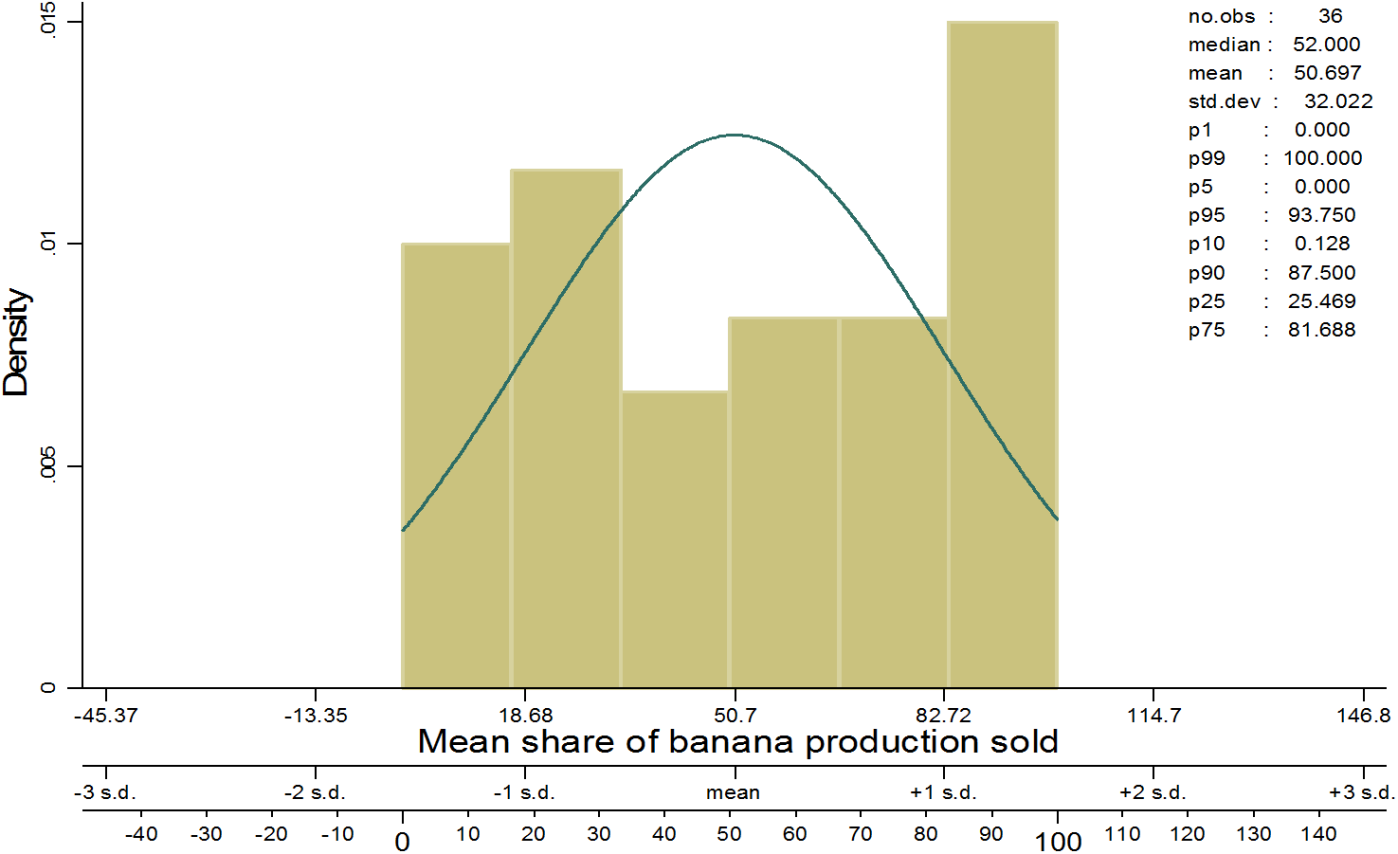
Proportion of annual banana production sold.

**Fig 6:**
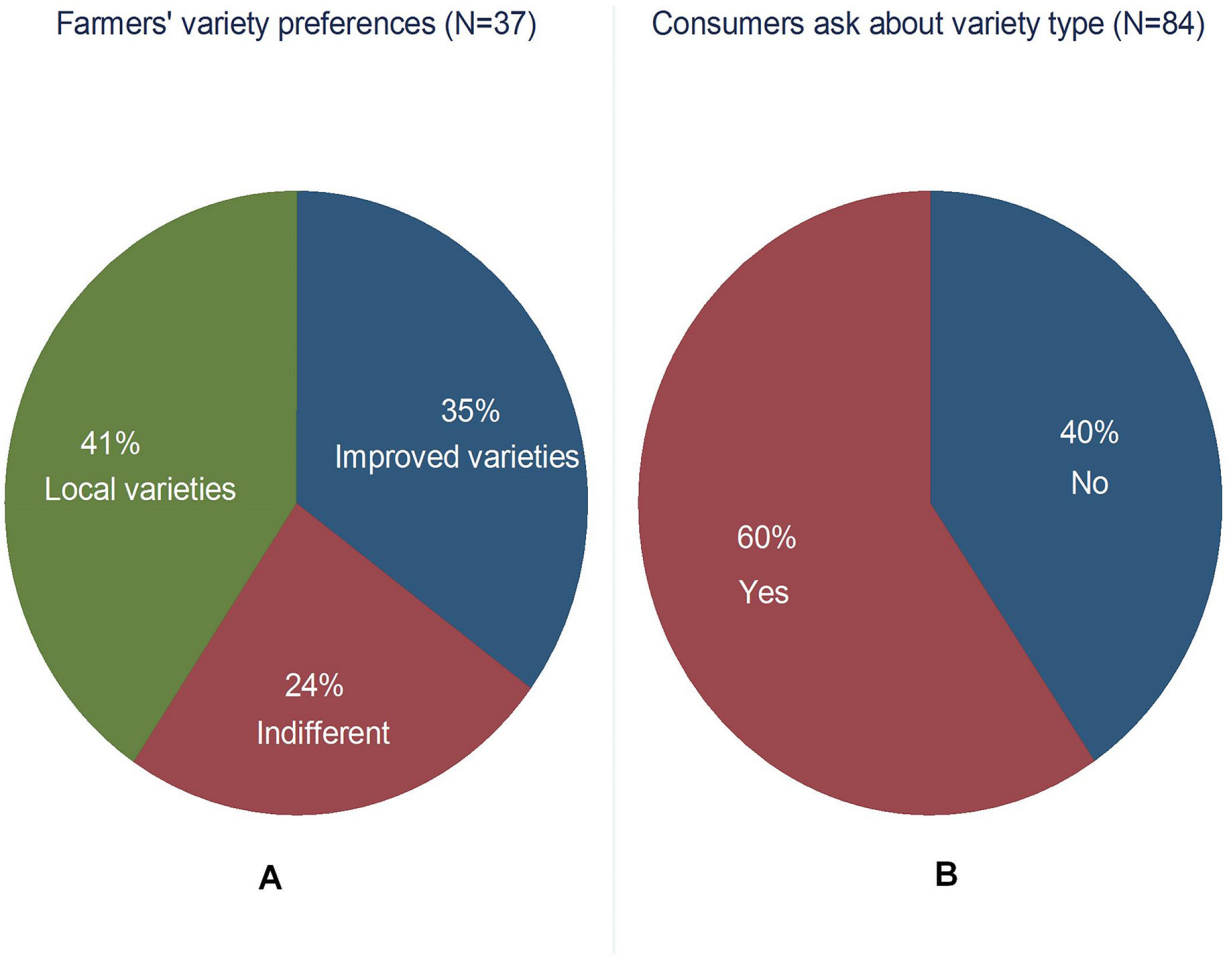
Farmers’ and consumers’ preferences and inquiries. (A) Represents farmers’ consumption preference for banana varieties. (B) Represents consumers’ inquiry about banana before purchase from either farmers or traders.

Among other factors, the adoption of new varieties is largely driven by consumer demand–both at the beginning of the supply chain (farmers) and at the end (net consumers). However, owing to limited resources, this study did not survey net consumers. Since banana production in the farming communities is used both for food and as a source of income, the consumers’ perceptions were indirectly deduced from those of the farmers. Nearly 50% of the total annual production is consumed on farm and the rest is sold (Fig 5). The potential consumption of GMB-BXW was tested against the existing experience of both farmers and traders with improved varieties. Fig 6A shows that 41% of the sample farmers prefer local to improved varieties because the former are tastier and easier to cook than the latter. On the flip side, for the same reasons, more than one-third (35%) preferred improved varieties, while nearly one-quarter found no differences between improved and local varieties in terms of taste and cooking time (Table 5).

**Table 5:** Preferences and consumption attributes between improved and local banana varieties.

Reasons for preferring local varieties to improved ones (N = 19)	**Percent**
Local varieties are tasty	84.2
Local varieties cook fast	15.8
Reasons for being indifferent between consuming local and improved varieties (N = 9)	
Both improved and local varieties are tasty	55.6
Both varieties cook fast	44.4
Attributes considered during purchase of bananas (N = 146)	
Quality and taste of banana	37.0
Size of the bunch and fingers	50.7
Banana prices	5.5
Good eye appeal	5.5
Long shelf life	1.3

The key question, however, asked was: What attributes do consumers consider when purchasing bananas? Both farmers and traders reported that more than half (60%) of their buyers asked about the type of the variety before purchase (Fig 6B). However, both traders and farmers stressed that the type of banana (local or improved) did not matter so much in influencing the decision whether to buy, but the size of the bunch and its fingers, the quality and taste mattered a lot (Table 5). These attributes are, thus, important to consider when new varieties are being developed.

### Potential consumers of GM banana resistant to BXW

**Fig 7:**
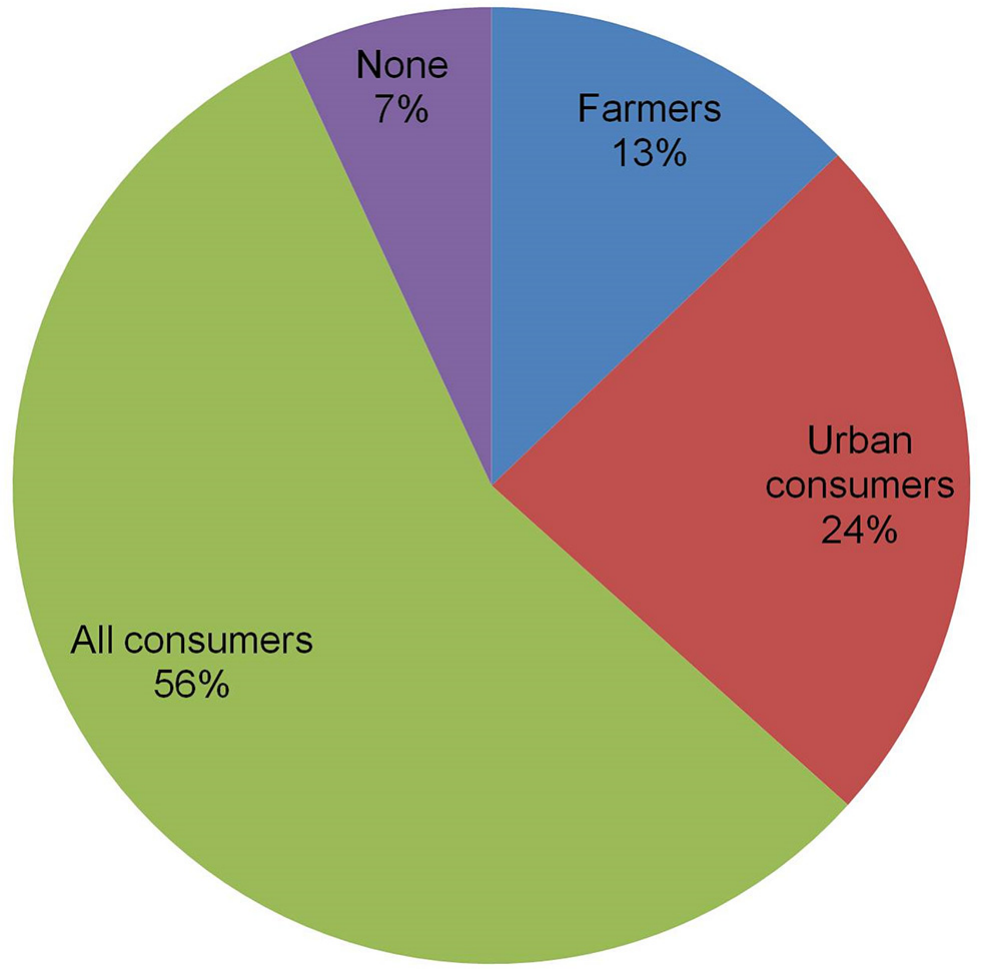
Potential consumers of GM banana (N = 101) in target countries.

**Fig 8:**
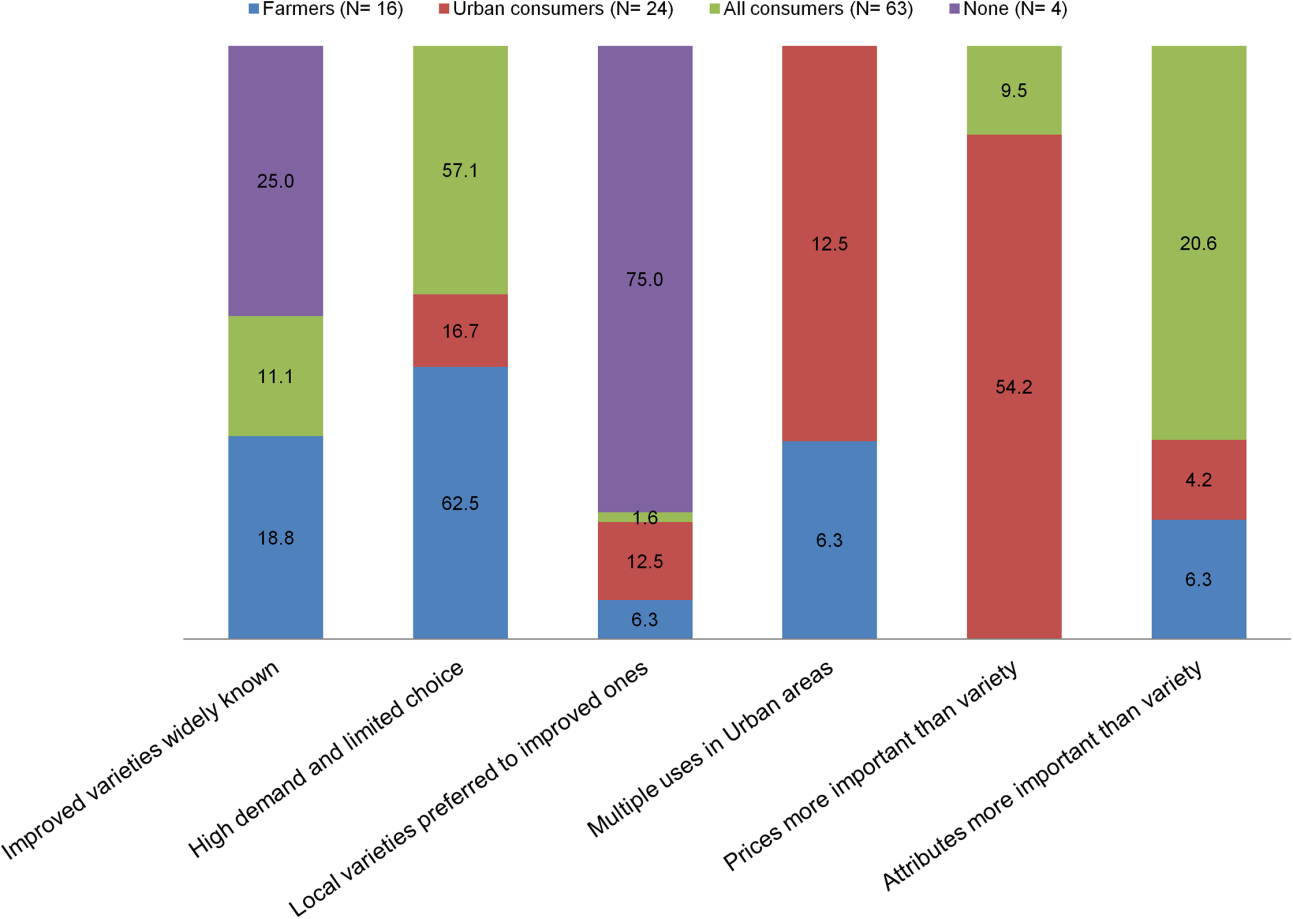
Frequency (%) of reasons for consumer preference of GM banana.

Against the background in the preceding section, farmers, traders, key informants and extension agents were asked to identify the potential consumers of GMB-BXW. More than half (56%) of the respondents, on account of the high market demand and limited choices, perceived that all consumers would not select against GMB-BXW but would be more concerned about consumption attributes than the type of variety (Figs 7 and 8). When urban and rural (farmers) consumers are compared, the former would be the main consumers (24% against 13%) as the price is more important to them than the variety. Very few (7%) would not consume GMB-BXW owing to perceived negative attitudes toward GM crops. These results further underscore the importance of preserving or enhancing the quality and taste of banana when breeding for disease and pest resistance.

### Economic evaluation of GMB-BXW development

Although a few studies have evaluated the economic impacts of BXW on banana production [26, 27, 28, 29], hardly any study has evaluated the potential adoption of GMB-BXW and its impact on both consumers and producers. Kalyebara et al. [30] showed that, in a period of 10–15 years, banana farmers in Uganda would lose US$5.6 billion if BXW remained uncontrolled and US$3.1 billion if the disease was controlled through removing the male bud and infected plants, using sterilized farm tools and uprooting the whole infected mat. The residual loss associated with existing control measures underscores the development of cultivars resistant to BXW. Similarly, Abele and Pillay [27] indicated that farmers would benefit from uncontrolled BXW only in the early years and lose in the later stages of BXW, whereas consumers would be worse off. More precisely, measured against the baseline, farmers would gain US$13 million from a 50% increase in prices; consumers would incur losses of US$160 million. Kayobyo et al. [31] predicted that uncontrolled BXW spreading at an annual infection rate of 8% would translate into an annual production loss of 2 million tonnes. This present paper complements these studies by going a step farther to estimate the economic benefits of GMB-BXW based on the survey data collected from key stakeholders.

Table 4 reports the survey results and assumptions to measure the ex-ante economic benefits of GMB-BXW using the ESM described above. The results (Table 4) are largely based on the survey data as described in the footnote to the table. The results show that farmers expect to increase the area allocated to banana cultivation by 10–50%, incur increased input costs by 20–50%, especially on acquiring GMB-BXW plantlets and establishing new plantations, and increase yields by 25–70% across target countries. The change in input costs also accounts for savings in labor costs from practices no longer needed to control the spread of BXW. The question was asked about reductions in the time spent in management practices to control BXW, and farmers indicated this labor requirement would be negligible in places where incidence of BXW was low. This is because some of the management practices such as removing suckers (sick plants) and male buds are part of the routine activities.

Other variables included in the ESM analysis are the minimum and ceiling rates of adoption. A uniform lag of 10 years was used across all countries to reach the ceiling adoption levels from the reported minimum adoption levels. This is because the diffusion of agricultural technologies often takes a relatively long time due to resource limitations for dissemination. The minimal variation may also be attributed to the small sample of respondents.

To obtain economic benefits of GMB-BXW at a national level requires information on the national banana cultivation area and production data. This information was obtained from FAOSTAT [18]. The farm gate prices were obtained from farmers’ surveys as an average price for each country. The total research costs of US$ 3,233,800 were based on the budget of the GMB-BXW project proposal for activities from 2013 to 2020. The costs include laboratory and screen house, confined field trials and food and environmental safety studies. The extension service costs during dissemination were assumed to be US$ 80 per hectare. The values for other parameters used in the ESM were based on available literature including, among others, Alston et al. [25]. These parameters included price elasticity of demand (0.5) and price elasticity of supply (1.0).

Table 6 reports measures of the ex-ante economic impacts of GMB-BXW in the GLA: the net present value (NPV) which reflects the net benefit of an investment in present value terms; the internal rate of return (IRR) which measures and compares the profitability of investments; and the benefit-cost ratio (BCR) which measures an investment’s benefits per unit cost. An ex-ante analysis of a 25-year period, including research and extension costs, and assuming a 10% discount rate, reveals that the NPV of the net benefits range from US$ 20 million–953 million in the target countries. The benefits are highest in Uganda (US$ 953 million), DRC (US$ 168 million) and Burundi (US$ 161 million). This is not surprising given that these same countries have faced and are still facing high yield losses from BXW. This suggests that investment in the development of GMB-BXW is not only essential but also economically viable. Certainly, returns on investments in research and extension are highest (56–86%) in these three countries compared with others in the region (30–43%). Finger et al. [12] found similar results in a number of countries producing GM crops. Correspondingly, the BCR estimates are high (17–34:1) in Uganda, Burundi and DRC compared with the rest of the target countries (4–21:1), indicating that investments in research and extension programs on GMB-BXW are feasible and have a great potential to generate a stream of benefits in excess of costs. Each dollar invested in the development and dissemination of GMB-BXW generates the highest amount in Burundi (US$ 34) followed by Uganda (US$ 30), Kenya (US$ 21), DRC (US$ 17), Tanzania (US$ 6) and Rwanda (US$ 4).

**Table 6:** Potential benefits of developing GMB-BXW in the GLA region.

Country	Burundi	DRC	Kenya	Rwanda	Tanzania	Uganda
Adoption ceiling area (‘000 ha)	117	361	36	244	567	1,354
NPV of gross consumer surplus (millions US$)	110	119	42	19	61	658
NPV of gross producer surplus (millions US$)	55	60	21	9	31	329
NPV of net total benefits (millions US$)	161	168	60	20	76	953
Internal rate of return (%)	56.48	57.79	43.21	29.6	43.14	85.64
Benefit–Cost ratio	33.85	17.13	20.71	3.62	6.11	30.05

Net Present Values (NPV) computed using a real interest rate of 10%.

Across all countries, benefits to consumers from the development and production of GMB-BXW would be twice as much as those accruing to farmers. For example, in Burundi the consumer surplus is US$ 110 million which is twice the producer surplus of US$ 55 million. This distribution of benefits is similar to those of the ex-ante analysis of GM ring spot virus–resistant papaya in Thailand [8] and the Philippines [32] that show consumers benefit twice as much as farmers.

## Sensitivity Analysis

**Fig 9:**
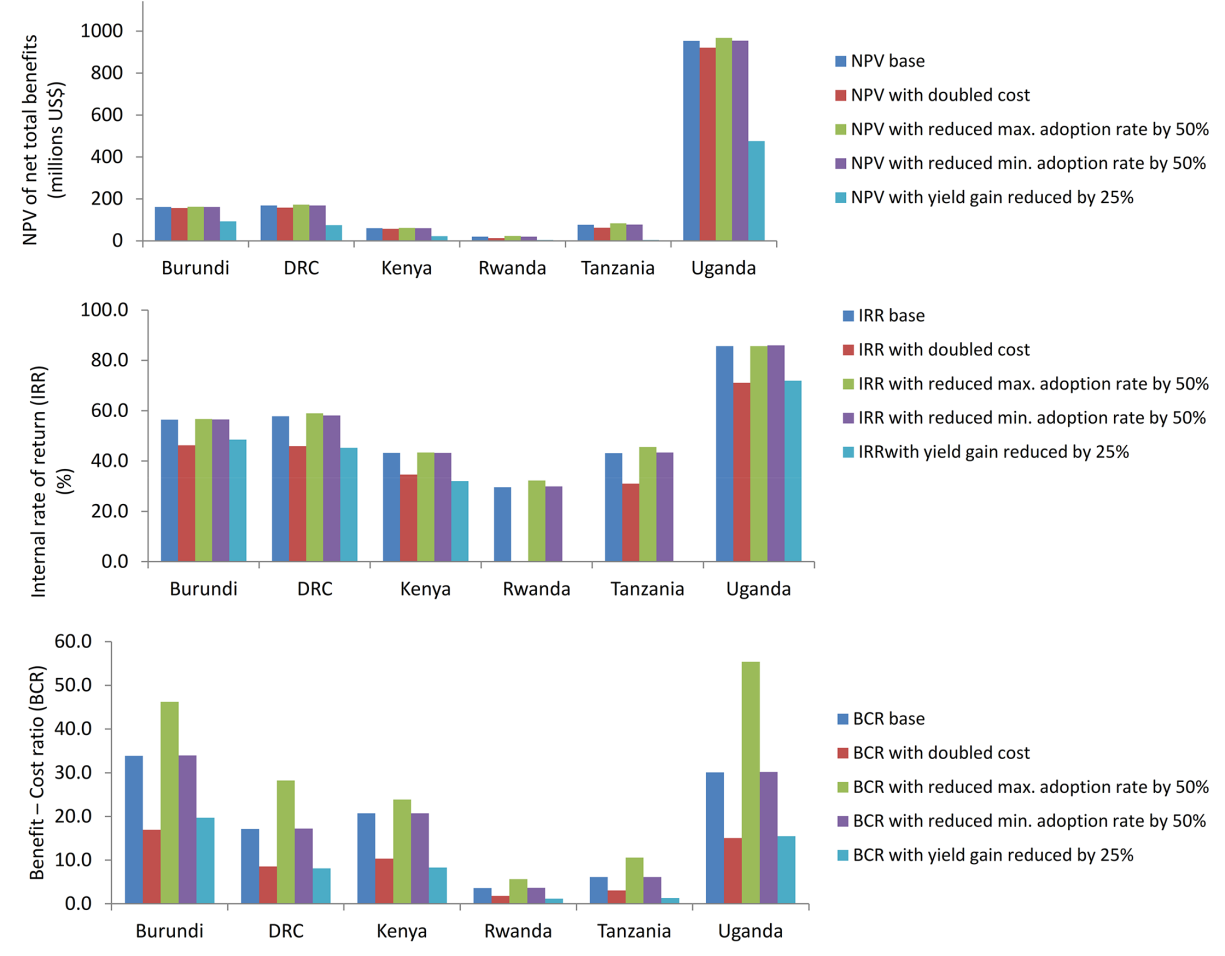
The upper panel reports the changes in NPV relative to baseline values (Table 6) due to doubling of inputs costs, reduction of initial and ceiling adoption rates by 50%, and reduction of yield by 25%. The middle panel reports the changes in IRR relative to baseline values (Table 6) due to doubling of inputs costs, reduction of initial and ceiling adoption rates by 50%, and reduction of yield by 25%. The bottom panel reports the changes in BCR relative to baseline values (Table 6) due to doubling of inputs costs, reduction of initial and ceiling adoption rates by 50%, and reduction of yield by 25%. Notes: In the upper, the yield gain for Rwanda was reduced by 5% as reduction by 25% would lead to economic losses. In the middle panel, the IRR for Rwanda could not be computed when input costs are doubled, while IRR for Tanzania could not be computed when yield gain was reduced by 25%.

Economic models often have key parameters that drive estimated results. Therefore, it is important to understand the robustness of model results by conducting sensitivity analysis with respect to key parameters. Sensitivity analysis was done to gain some insight about possible future changes in some of the parameters and their potential impact on the estimated economic benefits and costs of adopting GMB-BXW. The baseline results reported (Table 6) proved robust to some changes in the assumed levels of analyzed parameters reported in Fig 9. The projected increase in input costs because of adopting GMB-BXW was fairly low at 32% on average, while the initial and ceiling adoption rates were as high as up to 70% and 100%, respectively (Table 4). The expected yield gain after the adoption of GMB-BXW was also fairly high but with marked variability across countries, ranging from 25–70%. Holding the discount rate fixed at 10%, we doubled the total input costs, reduced the initial and ceiling adoption rates by 50%, and reduced the maximum yield gain by 25%, and then recalculated NPV, IRR and BCR. However, by reducing the maximum yield gain by 25% in Rwanda would lead to economic losses. We therefore reduced the maximum yield gain by 5% for this country. Also note that this reduction in yield gain (by 5% in Rwanda and 25% in Tanzania) could not allow computation of IRR for these countries. A similar problem was encountered in case of doubling costs for Rwanda. The upper panel of Fig 9 shows that changes in input costs or adoption rates have no significant impacts on NPV, suggesting that adoption of GMB-BXW remains economically viable despite increases in input costs or reductions in adoption rates. However, a reduction of 25% in the expected yield gain considerably reduces NPV, suggesting that yield improvement represents one of key factors affecting changes in net benefits and so is the potential likelihood of adopting GMB-BXW.

Doubling input costs and reducing yield gain by 25% would reduce IRR slightly while reductions in adoption rates would have no considerable impact on IRR (Fig 9, middle panel). The persistently high and positive IRR above the interest rates of most financial institutions and possibly the cost rate of donor investments reflects potential sizable returns to investment in the development of GMB-BXW. Regarding the changes in BCR, the bottom panel of Fig 9 shows that doubling input costs (or reducing yield gain by 25%) would reduce BCR by at least 50% but its value would remain positive, while reducing the ceiling adoption rates would increase BCR. The former suggests that investment in GMB-BXW development remains profitable even if input costs are more than doubled or expected yield gains are reduced by 25%; the latter suggests that the adoption of GMB-BXW is more profitable to early adopters than to late adopters when production occurs on a large scale. Finally, we also experimented with different discount rates by reducing and increasing the base discount rate of 10% by 50%. This is quite a significant change and the likelihood of it happening is very low. Results not reported but available from authors on request revealed that reducing (increasing) the discount rate by 50% would increase (reduce) the NPV values for each country reported in Table 6 by at least twice as much. This suggests that significant changes in economic environment and/or monetary policies such as interest rates may have serious implications on the benefits derived from the adoption of GMB-BXW.

## Conclusions

Banana production has been greatly affected by BXW in the GLA. The existing management practices to control the disease reduce the effects to only a small extent. If unchecked, BXW remains one of the biggest threats to banana production, which provides food security and income to millions of farm households in the GLA. To overcome these threatening effects, scientists have successfully developed a GM banana resistant to BXW. Although GM crops have been developed and proved economically beneficial in other countries, the development of GM crops in the GLA is still in its infant stages. On the one hand, the government policies legalizing the release and commercialization of GM crops are still under formulation although some countries have allowed research on GM crops to take place. Only Kenya in the GLA has a biosafety law and a national Biotechnology and Biosafety policy; Uganda has a national Biotechnology and Biosafety policy and the formulation of biosafety laws is in advanced stages of the policy cycle. The discussion of biosafety laws and policies in other countries in the region has not yet gained enough momentum. On the other hand, studies to assess the ex-ante adoption and potential benefits are still limited in the region. This study provides evidence on the potential economic importance of GM BXW-resistant banana to be released in the GLA. The evidence is based on data collected from farmers, traders, extension agents and key informants from Burundi, DRC, Kenya, Rwanda, Tanzania, and Uganda.

The results show that farmers would be willing to adopt the GM BXW-resistant banana when released. The initial adoption rate ranges from 21 to 70% and would reach the adoption ceiling of up to 100% in 2–10 years. The findings further show that if this new technology were successfully adopted in the GLA, both consumers and producers would benefit. However, the former would benefit twice as much as the latter owing to the price reduction from the excess, stable and continuous supply of banana. The magnitudes of benefits vary considerably across countries. Largest benefits would accrue to consumers and farmers in countries that have had and are experiencing large production losses from BXW. Uganda, Burundi and DRC would receive the largest benefits ranging from US$ 161 million to 953 million compared to US$ 20 million to 76 million in the other countries. The computed IRR is very high in each study country (between 29 and 85%), above any known interest rate in the banking institutions, and provides additional evidence of the profitability of investing in GM BXW-resistant banana.

The study reveals implications for the research scientists and extension agents, as they are frequently interested in knowing the important triggers of successful adoption and consumption of new technologies. The results in this paper not only demonstrate that investment in the development of GM BXW-resistant banana is viable, but also provide a basis on which to focus research and extension efforts on the attributes that would significantly enhance adoption of new banana varieties. Despite the interesting findings reported in this article, which are consistent with and supported by the existing literature, limited resources prevented us the use of large sample sizes and consumer surveys. Future studies of a similar nature can include consumer surveys in addition to all stakeholders along the value chain as well as utilizing the advantages of large sample sizes.

## Supporting Information

S1 Dataset The zipped folder contains different data files. The files contain data used to generate results reported in the paper for different respondent categories (farmers, extension agents, traders and key informants). There are also files which contain pooled data from different respondent categories. The pooled datasets specify which tables or results were generated from them. The folder also contains a “Read me” file with more information about the data.
(RAR)
